# *You can’t see what you don’t measure!* A scoping review of measurements of gender-based violence, its determinants and consequences in academia

**DOI:** 10.1371/journal.pone.0317872

**Published:** 2025-02-13

**Authors:** Claudia Schredl, Anke Lipinsky, Anne Laure Humbert

**Affiliations:** 1 Central Gender Equality Office, University of Cologne, Cologne, Germany; 2 Department Data and Research on Society, GESIS - Leibniz Institute for the Social Sciences, Cologne, Germany; 3 Department of Sociology and Work Science, University of Gothenburg, Gothenburg, Sweden; National Institute of Public Health: Instituto Nacional de Salud Publica, MEXICO

## Abstract

**Introduction:**

Assessing the problem of gender-based violence in academia internationally is challenging due to a lack of empirical evidence and differences in how it is measured. The contribution of this article is to reflect on survey measurements and to propose new avenues for future quantitative measurements of gender-based violence, its determinants, and consequences in an academic context. For this purpose, we present the results of a scoping review of ten national and cross-national prevalence studies on gender-based violence. We examined the studies’ quantitative operationalisation of (1) sex and gender, (2) prevalence of gender-based violence, (3) socio-demographic determinants from an intersectional perspective, individual and contextual factors, (4) and consequences.

**Materials and methods:**

Prevalence studies were identified through a comprehensive search of electronic databases and specialised data repositories. The selection criteria included studies with a focus on gender-based violence, the use of closed-ended survey questions, i.e., in a questionnaire, the potential for applicability in different national contexts, and the specific context of higher education. Eligible sources also described the quantitative operationalisation of the survey measurements.

**Results:**

Our work critically reviews previous efforts to measure gender-based violence, its determinants, and consequences in academic contexts. The findings of our assessment show, first, that quantitative gender-related measurements tend to conflate the concepts of sex and gender, and hence their operationalisation in quantitative surveys. Second, there is a strong emphasis on sexual harassment and sexual violence to the detriment of other relevant forms of violence, as well as a rooting of measurement concepts in locally valid but diverse legal definitions of gender-based violence. Third, there is a lack of socio-demographic determinants to provide an intersectional lens, as well as a focus on measurement frameworks that individualise the experiences of gender-based violence. This prevents a conceptualisation of harassment and abuse as a structural problem in the academic sector and ignores that violence is both as a cause and a consequence of unbalanced gendered power relations and inequalities in institutional and societal contexts. Fourth, there is generally a retrospective approach to measuring the consequences of gender-based violence, which may represent a source of potential bias. We reflect on how future survey instruments could address these issues in academic environments.

**Discussion:**

Overall, our paper demonstrates how the evidence generated by different conceptualisations and operationalisations of gender-based violence in academia, as well as its determinants and consequences, shapes how we think and talk about the problem. In doing so, this article contributes to the ongoing methodological discussion on the measurement of gender-based violence and provides a rationale for improving its measurement framework in academic environments by taking a feminist-theory informed approach to collecting these data.

## Introduction

Despite growing policy interest in gender-based violence in academia in recent years, it remains an underdeveloped field of knowledge in Europe [1]. Gender-based violence is a complex, persistent and often unspoken feature and force in many organisations, including universities and research organisations [2,3] with serious, sometimes fatal, consequences [4,5]. Gender-based violence is a broad and evolving concept. It may encompass many different forms of aggression, harassment, abuse, or incivility, unified by an understanding that these different forms are both causes and consequences of gender power inequalities. Our understanding of violence is based on a feminist perspective that allows for plurality and specificity. What constitutes violence lies in the experience of those affected and is not limited to a legally defined framing of violence [6]. Violence is gender-based in that it affects all genders: women, men, and people identifying as non-binary in different ways. It should always be acknowledged that gendered relations of power typically imply a cis-heteronormative hierarchy in which women and non-binary people are disproportionately affected by violence [7,8].

It is difficult to understand the scope of the problem of gender-based violence [9], including its consequences in academia, due to a dearth of comparable evidence as few empirical studies exist that allow comparison across different higher education contexts e.g., [10–12]. Most prevalence studies focus on sexualised forms of gender-based violence, such as sexual harassment and sexual violence. Data on the behaviour of bystanders or perpetrators are also rare. Many empirical studies collect data in one country e.g., [13], often in a single institution e.g., [14,15] and apply a focused approach to specific groups, e.g., by targeting students or women exclusively. Data are collected for different purposes, such as to inform institutional policy making. It is therefore not surprising that a variety of concepts and measurements are used today [16]. Previous quantitative studies tend to leave out contextual information that are needed to interpret prevalence rates, such as the level of organisational tolerance for violence and harassment or the normalisation of violence. Including this information in the measurement framework is useful to avoid an individualising understanding of gender-based violence. Finally, many existing studies do not collect any data on consequences for victims/survivors or include only a few retrospective questions.

This lack of consistent measurement frameworks hinders cross-cultural comparisons in the European Research Area (ERA) and impedes progress toward supra-national evidence-based interventions. To address this gap, we implemented a scoping review of prevalence studies in academia. By systematically mapping existing measurements of gender-based violence, including its determinants and consequences, we aimed at identifying measurement biases and gaps.

In this scoping review, we demonstrate how prior studies have tended to 1) conflate the concepts of sex and gender, 2) focus on measuring sexual violence and harassment, and thereby neglecting other interrelated forms of gender-based violence, 3) individualise the problem of gender-based violence rather than seeing it as structural mechanism to maintain existing inequalities, and 4) take a retrospective approach to collecting data on the consequences of gender-based violence – if they measure them at all.

We also show how different conceptualisations of ‘violence’ and ‘gender’ interrelate and have shaped previous data collection, posing challenges in their practical implementation within survey measurement frameworks, as well as how we think and talk about gender-based violence in academia, including its prevalence. Our paper contributes to reflections on the knowledge base on gender-based violence in academia by providing a detailed overview of how measurements of different forms of gender-based violence, its determinants and consequences have been operationalised in previous surveys. This comprehensive endeavour seeks to enhance our understanding of gender-based violence in academia and inform quantitative survey research and policy efforts in the future.

## Materials and methods

**Fig 1:**
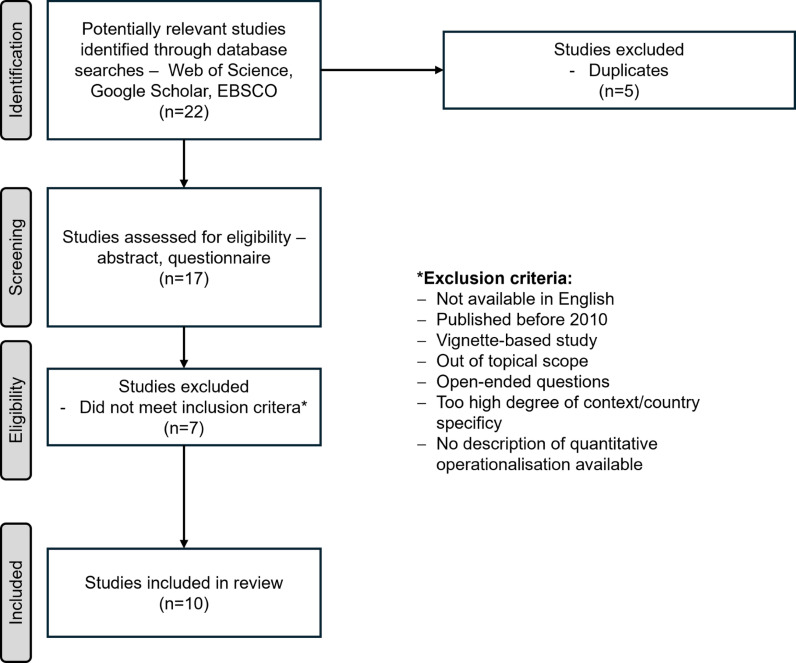
Study selection process.

Prevalence studies on gender-based violence, its determinants, and consequences were identified through a comprehensive search of various electronic databases, including the ERIC Institute of Education Sciences database, the CESSDA Data Catalogue, Web of Science, Google Scholar, EBSCO, and Scopus, as well as specialised data repositories such as the Measurement Instrument Database for the Social Sciences, Social Science Open Access Repository, European Social Survey, Inter-university Consortium for Political and Social Research, and Harvard Dataverse. Fig 1 outlines the study selection process in more detail.

The UniSAFE theoretical framework [6] was used to deduct key search topics and terms. The search terms and phrases from the theoretical framework were supplemented by synonyms, related terms, generic and subordinate terms, different spellings in English and then searched in addition or in combination (*AND * , or * OR*) with faceting terms and phrases. For example, a combination of terms and phrases was used to capture different forms of violence, including, ‘gender-based violence’, ‘violence against women’, ‘intimate-partner violence’, ‘GBV’, ‘IPV’, ‘rape’, ‘harassment’, ‘sexual harassment’, and ‘gender harassment’. We then added terms from specific contexts in which they should occur, including, for example, ‘academia’, ‘higher education’, and ‘universities’. The following search string is provided as an illustration: sexual harass* ” OR (harass* AND sex*) AND (universit* OR college* OR academ* OR “higher education” OR work* OR organisation*) NOT (child* OR school*).

The selection criteria are based on the research design used to develop a questionnaire for the large-scale cross-cultural online survey of the UniSAFE project, which focused on gender-based violence, its determinants, and consequences in academia. For practical reasons, such as translation costs and time constraints, we excluded studies not available in English, vignette-based studies, prevalence studies published before 2010, or studies using open-ended questions. Eligible sources also described the quantitative operationalisation of the survey measurements of sex and gender, prevalence of gender-based violence, determinants, individual and contextual factors, and consequences.

To adequately address our scope of research on prevalence and consequences of gender-based violence in academia, we included prevalence studies that were conducted among university members, including university students and/or university staff. Nine of the ten selected studies focused on the prevalence and consequences of gender-based violence in academia. We made an exception for the FRA study, which is the largest EU-wide survey on violence against women to date. Finally, we assessed the conceptual transferability of the studies’ individual measures to different countries (i.e., the degree of context/country specificity). For example, whether the prevalence question on sexual violence was asked on the basis of country-specific legal definitions of ‘rape’ or whether it was more comprehensive and therefore applicable to different national contexts.

The ten studies selected for the scoping review are presented in Table 1. From these studies, we examined the operationalisations of (1) sex and gender, (2) prevalence of gender-based violence, (3) determinants, individual and contextual factors, as well as (4) consequences of gender-based violence. We developed guideline questions on the conceptualisation and operationalisation of the measurement instruments for each of the four categories. Data were extracted for each study and transferred into an Excel file, with one table per category. The tables included the original question and response options in addition to the original ID, the source study of the item, the latent measurement concept, and metadata on the question format. The measurement instruments of the ten studies were analysed qualitatively from a feminist standpoint theory perspective [17,18]. All authors reviewed and discussed the results. Through an iterative process of reviewing and synthesising the data, consensus was reached on the most essential findings. A consistent component of the analysis was the comparison of the measurements in terms of form and content. The qualitative content analysis revealed similarities and differences between the operationalisations and conceptualisation of sex and gender, prevalence of gender-based violence, determinants, individual and contextual factors, and consequences.

**Table 1:** Studies that include measurements of gender-based violence, its determinants and consequences selected for the scoping review.

ID	Study reference	Name	Issuing organisation	Target group	Geographic coverage	Measurements included in the study
1	AAU, 2015 [12]	AAU Campus Climate Survey on Sexual Assault and Sexual Misconduct	AAU - Association of American Universities	University students 18+	United States	Includes measures of experiences of sexual assault and misconduct of students, with a focus on a university context, takes communication technologies and intimate partner violence into account. Also includes questions directed to bystanders. Measures knowledge about support services, seeking support and reporting by victims and bystanders.
2	AHRC, 2017 [13]	Change the course: National report on sexual assault and sexual harassment at Australian universities	AHRC - Australian Human Rights Commission	University students 18+	Australia	Includes measures of experiences sexual assault and sexual harassment of students, with a focus on the university context, takes communication technologies into account. Also includes questions directed to bystanders. Measures knowledge about support services, seeking support and reporting by victims and bystanders.
3	ARC3, 2015 [19,20]	ARC3 Campus Climate Survey	ARC3 – Administrator Researcher Campus Climate Collaborative	University staff and students	United States	Includes measures of experiences of physical violence, sexual violence, and sexual harassment of staff and students, including information on institutional responses and individual attitudes (e.g., peer norms).
4	FRA, 2014 [21]	Violence against women: an EU-wide survey	FRA – European Union Agency for Fundamental Rights	Women, aged 18–74	EU Member States	Includes measures of experiences of physical and sexual violence, sexual harassment of adult women, including stalking and taking communication technology into consideration. All measures for intimate partners and non-partners, also questions about seeking support and reporting by victims.
5	Gendercrime, 2011 [11]	Gender-based Violence, Stalking and Fear of Crime (Gendercrime)	Ruhr-Universität Bochum (RUB)	Female higher education students	Germany, Italy, Poland, United Kingdom, and Spain	Includes measures of experiences of sexual violence and harassment of female higher education students in five (then) EU countries. Also asks about knowledge about provision of institutional support services.
6	HEA, 2021 [22,23]	National Survey of Student Experiences of Sexual Violence and Harassment in Irish HEIs	HEA - Irish Higher Education Authority	University staff and students	Ireland	Includes measures of experiences of sexual violence and harassment of university staff and students of Irish higher education institutions, including information on contextual factors and individual attitudes (e.g., bystander attitudes, and individual attitudes to and perceptions of sexual violence and harassment).
7	KI, 2022 [10]	Gender and sexual harassment in Higher Education in Sweden	KI - Karolinska Institutet;	University staff, PhD students, and students	Sweden	Includes measures of experiences of diverse forms of gender-based violence from a victim, bystander, and perpetrator perspective, including questions about reporting/seeking support and the relationship between those actors. Communication technologies are considered. Also asks about the work/study environment and personal (mental) health.
8	NUS, 2018 [24]	Power in the academy: staff sexual misconduct in UK higher education	NUS - National Union of Students (in conjunction with The 1752 Group)	(former) higher education students 16+	United Kingdom	Includes measures of experiences of sexual misconduct of staff towards students from the perspective of victim and bystander, and their consequences, the reporting of incidents, as well as the perceived appropriateness of certain behaviours.
9	UCU, 2021 [14]	Sexual violence UCU member survey	UCU - University and College Union	Staff only	United Kingdom	Includes measures of experiences of gender-based violence of staff. Victim and bystander perspective considered as well as bystander interventions. Also asks about organisational responses and reactions towards gender-based violence as well as prevention and support services provided.
10	Wits, 2018 [15]	Survey on gender-based harm at the University of the Witwatersrand	Wits - University of the Witwatersrand	University staff and students 18+	South Africa	Includes measures of experiences of different forms of gender-based violence of university staff and students, including information about the perpetrator, the consequences of gender-based violence on work and studies, perceptions of support, and prosecution of gender-based violence, attitude questions about gender, relationships, sex, and LGBTQ categories.

Source: [25,26].

This study has been reported according to the PRISMA Checklist (S1 Checklist PRISMA-ScR).

## Results

The scoping review indicates that most measurement frameworks of gender-based violence in an academic context demonstrate a narrow understanding of prevalence. This is manifested in several ways, including adopting a strong focus on sexualised forms of gender-based violence, such as sexual harassment and sexual violence; by restricting the studies’ scope to ciswomen only; by not capturing the perspectives of bystanders and perpetrators; and/or by not taking into account socio-demographic determinants from an intersectional perspective, individual and contextual factors, or the consequences of gender-based violence. This section presents the results of the scoping review of the studies’ quantitative operationalisation of the following: (1) sex and gender, (2) prevalence of gender-based violence, (3) socio-demographic determinants from an intersectional perspective, individual and contextual factors, and (4) consequences.

### Sex and gender

The scoping review of concepts and operationalisations of sex or gender indicates a conflation between sex and gender in most of the measurements (Table 2). While gender theory attempts to do justice to the complexity, fluidity, interconnectedness and overlap of gender concepts, quantitative survey research makes concessions in that it resorts to computable classification and categorisation schemes. This makes it all the more important that the operationalisation of gender constructs in quantitative studies are selected in an informed manner, i.e., taking into account relevant gender theory considerations in the measurement design [18,27]. A conflation of concepts may be indicated, for example, when the question asks about gender, but provides answer categories related to sex (i.e., female, or male). This conflates two interrelated concepts: ‘gender’ (as socially constructed roles, behaviours, expectations, and societal norms that societies consider appropriate for people) and ‘sex’ (based on biological, physiological, or legal assignments that define humans as female or male) [28]. Of the studies included in the review, only one (HEA) differentiates between the concepts of sex and gender in the survey questions, inquiring about both the respondents’ sex and gender.

**Table 2:** Existing measurements of sex and gender.

ID	Study	Conflation between sex and gender	Non-binary approach	Different order of answer categories for gender-variables of survivors and perpetrators
1	AAU, 2015	No	Yes	Yes
2	AHRC, 2017	Yes	Yes	No
3	ARC3, 2015	No	Yes	Yes
4	FRA, 2014	^a^	No	^a^
5	Gendercrime, 2011	Yes	No	Yes
6	HEA, 2021	No	Yes	Yes
7	KI, 2022	Yes	Yes	No
8	NUS, 2018	No	Yes	No
9	UCU, 2021	No	Yes	^b^
10	Wits, 2018	Yes	Yes	Yes

^a^No question on respondents’ sex/gender because only women were interviewed.

^b^Perpetrator’s gender not asked.

Eight out of the ten studies (AAU, ARC3, ARHC, HEA, NUS, KI, UCU, Wits) include more than two categories when asking respondents about sex or gender as socio-demographic variable. A non-binary approach to operationalising ‘gender’ recognises that gender identities and expressions exist on a spectrum, rather than being confined to a binary understanding of women and men. For example, non-binary, genderqueer, or genderfluid individuals may not identify strictly as women or men and may embrace a range of gender identities and expressions or reject the gender binary altogether. A non-binary approach to operationalising ‘sex’ is also sensitive to the existence of intersex people and people who reject sex categorisation [18]. However, the non-binary response categories provided differ among the studies and demonstrate the different approaches and challenges in categorising gender beyond the binary for quantitative analysis in social surveys (for an overview of survey measure of sex and gender, see [29–31]). Three of the eight studies (HEA, NUS, UCU) also ask whether the current gender identity of the respondent is the same as their sex assigned at birth, hereby aiming to capture people who identify as transgender.

The measurement of sex and gender is not limited to respondents only, but also extends to perpetrators in most studies. The UCU study is the only study which does not include a follow-up question on the perpetrator’s gender. Where studies do ask about the gender of both victim and perpetrator, the order of the gender categories often reflect the implicit assumption that the victim is a woman, and the perpetrator is a man. In five of the eight studies (AAU, ARC3, Gendercrime, HEA, Wits), that ask for the gender of both victim and perpetrator, the first non-randomised response option for victims is ‘woman’ or ‘female’, while for perpetrators the order is reversed, and the first response option is ‘man’ or ‘male’. Only three of the studies (AHRC, KI, NUS) maintained the same order of items.

Among the nine studies that measure the gender of perpetrators, seven adopt a non-binary approach to the gender of victims (AAU, AHRC, ARC3, HEA, KI, NUS, Wits). However, only five studies (AAU, ARC3, HEA, KI; NUS) extend the non-binary approach to perpetrators. This may reflect the difficulty of responding to questions about gender when it comes to perpetrators in victim-centred prevalence surveys. While responding to a question on gender identity is a self-identification of the respondent, the gender of the perpetrator(s) is ‘read’ by the respondent, i.e., interpreted or perceived based on visible or assumed characteristics, such as appearance, behaviour, voice, or clothing, often aligning with societal norms or stereotypes. This process can lead to assumptions that may not align with the perpetrator’s actual gender identity. Invariably, in the case of a single perpetrator, it is only possible for the respondent to read the gender of the perpetrator. In the case of multiple perpetrators, the precise gender composition might be even more difficult to read. Nonetheless, it remains important to seek this information – no matter how imperfect the measurement might be – to better understand the gendered power dynamics of the incidents of violence that are measured in a specific context such as academia.

Our analysis reveals the underlying conceptual understanding of gender-based violence in previous studies, which implicitly define men as perpetrators and women as victims of gender-based violence. Women and minoritised gender groups are generally subordinated, marginalised and the expected majority of victims of gender-based violence [13]. The implicit cis-heteronormative understanding of the relationship between perpetrator and victim permeates the measurements with a focus on certain forms of gender-based violence, such as sexual harassment and sexual violence.

While recognising gender asymmetry in the experience of gender-based violence [9,21,32] and gender inequalities in power relations that disadvantage women [33], we argue for a holistic understanding of gender in quantitative prevalence studies that recognises and analyses the experiences of all gender identity groups [34], and examines the gendered relationship of victim(s)-perpetrator(s) in a given context.

### Prevalence of gender-based violence

Sexual harassment and sexual violence are the most researched forms of gender-based violence, particularly in academia. Out of the ten studies, only four (AAU, ARC3, FRA, KI) go beyond sexual harassment (including stalking) and sexual violence (see Table 3). All four include questions about incidents of online violence separately from experiences of other forms of gender-based violence (which can also take place online), thus taking into account the role of digital technologies. These four studies (AAU, ARC3, FRA, KI) also asked the respondents about their experiences of physical and psychological violence. However, some of the forms of violence are only examined in the context of intimate partner violence. Both, the AAU and the ARC3 survey examine the prevalence of physical and psychological violence only in the context of intimate partner violence. In the ARC3 survey, the items on physical and psychological violence are also referred to as ‘dating violence’. The FRA survey also asked respondents about their experience of physical violence with persons other than their current or former partner, although all questions on psychological violence refer to intimate partner violence. The KI survey is the only one that addresses physical and psychological violence outside the context of intimate partner violence, by asking the respondents whether they experienced these incidents in their professional role in academia. Our review findings indicate that little is known so far about how incidents of physical and psychological gender-based violence are located within the context of universities and research organisations.

**Table 3:** Existing measurements of prevalence of gender-based violence.

ID	Study	Target group	Number of gender-based violence forms measured	Forms of gender-based violence measured	Online violence measurement	Forms of gender-based violence measured in the context of intimate partner violence	Sources used to define gender-based violence	Information provided on the victim in relation to the perpetrator
1	AAU, 2015	University students 18+	6	Physical violence, psychological violence, sexual violence, sexual harassment, online violence, stalking	Separate question on online violence (part of stalking item scale) and online place of incident of sexual harassment	Physical violence, psychological violence	Legal definitions, previous studies on sexual harassment or gender-based violence	Victim-perpetrator relationship and position of perpetrator
2	AHRC, 2017	University students 18+	2	Sexual violence, sexual harassment	Online place of incident of sexual harassment	None	Legal definitions	Position of perpetrator
3	ARC3, 2015	University staff and students	4	Physical violence (including one item on psychological violence and one item on economic violence), sexual violence, sexual harassment, stalking (including items on online violence)	Separate items on online violence (part of stalking item scale)	Physical violence (including one item on psychological violence and one item on economic violence)	Source of definition unclear	Position of perpetrator
4	FRA, 2014	Women, aged 18–74	7	Physical violence, psychological violence, economic violence, sexual violence, sexual harassment, online violence, stalking	Separate question on online violence and online place of incident of sexual harassment	Psychological violence (including two items that can be considered as economic violence)	International definitions	Victim-perpetrator relationship
5	Gendercrime, 2011	Female higher education students	3	Sexual violence, sexual harassment, stalking (including items on physical violence, psychological violence, economic violence)	Online place of incident of stalking and sexual harassment	None	Theoretical concepts, international definitions	Victim-perpetrator relationship and position of perpetrator
6	HEA, 2021	University staff and students	2	Sexual violence, sexual harassment	Online place of incident of sexual harassment	None	Legal definitions, previous studies on sexual harassment or gender-based violence	Victim-perpetrator relationship and position of perpetrator
7	KI, 2022	University staff, PhD students, and students	5	Physical violence (not included in students’ questionnaire), psychological violence, sexual violence, sexual harassment, online violence	Separate question on online violence and online place of incident of sexual harassment	None	Previous studies on sexual harassment or gender-based violence	Victim-perpetrator relationship and position of perpetrator
8	NUS, 2018	(Former) higher education students 16+	2	Sexual violence, sexual harassment	Online violence not measured	None	Theoretical concepts, previous studies on sexual harassment or gender-based violence	Victim-perpetrator relationship and position of perpetrator
9	UCU, 2021	University staff only	2	Sexual violence, sexual harassment	Online violence not measured	None	Legal definitions, theoretical concepts	Position of perpetrator
10	Wits, 2018	University staff and students 18+	3	Physical violence, sexual violence, sexual harassment	Online violence not measured	Physical violence	Theoretical concepts, international definitions	Victim-perpetrator relationship and position of perpetrator

Economic violence, which includes acts or behaviours that cause economic or financial harm to an individual [35], is a form of gender-based violence which has not yet been included in prevalence studies in academia. However, in the FRA survey, two of the psychological violence items on intimate partner violence can be considered economic violence, including: preventing a woman from making decisions about family finances and from shopping independently; and forbidding her to work outside the home. As outlined in the FRA study description, the Istanbul Convention’s (Article 3) explanatory report ‘notes that economic violence or harm – which, for example, the Council of Europe or the United Nations have not separately addressed in some of the earlier definitions – can be related to psychological violence’ [13], p. 76.

Definitions of gender-based violence rely on legal definitions, international policy definitions, or previous studies, with theoretical concepts rarely discussed. Only four of the ten studies (Gendercrime, NUS, UCU, Wits) refer to theoretical concepts for defining gender-based violence. Instead, more than half of the studies draw upon either local legal definitions of sexual harassment and sexual violence (including sexual assault, sexual misconduct, etc.), definitions of gender-based violence used in previous studies, or international definitions.

The two most commonly cited international policy definitions of gender-based violence are the Council of Europe Convention on Preventing and Combating Violence Against Women and Domestic Violence (Istanbul Convention) adopted in 2011 [36], and the United Nations General Assembly (1993) Declaration on the Elimination of Violence Against Women [37].

The Istanbul Convention [36] defines the term ‘violence against women’ (Article 3) as follows:

*“(a) ‘violence against women’ is understood as a violation of human rights and a form of discrimination against women and shall mean all acts of gender-based violence that result in, or are likely to result in, physical, sexual, psychological or economic harm or suffering to women, including threats of such acts, coercion or arbitrary deprivation of liberty, whether occurring in public or in private life;”*

In the Declaration on the Elimination of Violence Against Women (Article 1) [37], gender-based violence is defined as:

*“Any act of gender-based violence that results in, or is likely to result in, physical, sexual, or psychological harm or suffering to women, including threats of such acts, coercion or arbitrary deprivation of liberty, whether occurring in public or in private life.”*

In order to allow for a non-binary gender approach, the Wits study adapted the definition of gender-based violence of the United Nations General Assembly (1993) Declaration on the Elimination of Violence Against Women [37], replacing violence against women with violence against all genders.

Looking more closely at the legal definitions of sexual harassment and sexual violence used of previous studies (AAU, AHRC, HEA), it becomes clear that there are wide variations in national and regional legal definitions of what constitutes unlawful behaviour. On the one hand, these differences make it difficult to harmonise the results of operationalisations of instruments used to collect data on the prevalence of sexual harassment and sexual violence across different national contexts, as the data collected should fit local legal and policy needs. On the other hand, this may also influence the respondents’ perceptions of what behaviour is morally wrong or against the law. As the AHRC study points out, ‘sexual assault has a specific meaning when used to describe particular criminal sexual offences, however, it also has a broader, more general meaning when used in everyday conversation’ [10], p. 27.

To address this issue, for example, the FRA survey did not refer to any legal definition when phrasing prevalence questions about sexual violence, sexual harassment, or other forms of gender-based violence. Considering that, despite the differences in the legal definitions across different contexts and countries, similar behaviour is often subsumed under the umbrella terms ‘sexual harassment’ and ‘sexual misconduct/assault’, presenting no explicit legal context may also be a legitimate approach for future cross-national surveys on gender-based violence.

Four out of the ten studies (Gendercrime, NUS, UCU, Wits) referred to theoretical concepts in the definition of gender-based violence. These included theoretical concepts of specific forms of gender-based violence, power abuse in academia, and at a more abstract level, the prerequisites of inequality and violence. For example, both the NUS and UCU surveys draw on Kelly’s [38] work to define sexual misconduct as ‘a continuum of behaviours that includes but is not limited to sexualised comments, sexual harassment, grooming, sexual assault, sexual coercion and control, and sexual violence’ (p. 41). In addition, the UCU study also refers to Kelly [38] recognising that sexual misconduct ‘can affect the survivor either at the moment of the offence or later “as a threat, invasion or assault, that has the effect of hurting […] or degrading [them] and/or takes away [their] ability to control intimate contact”’ [11], p. 10–11.

The ‘grooming process’ concept by Brackenridge [39] and Brackenridge & Fasting [40], which describes how perpetrators manipulate a potential victim to make them less likely to reject or report violence, is also discussed in the NUS study, as is the importance of critically investigating ‘notions of wanted behaviour and consensual relations’ in the context of unequal power relationships between staff and students as pointed out by Page et al. [41].

The Wits study refers to a theoretical concept of academic sexual harassment [42], which emphasises the use of authority in the context of sexual harassment and identifies students as targets of sexual harassment. In addition, the concept of contra-power sexual harassment [43–45] is discussed in the Wits study, arguing that also persons of higher status can be targets of harassment and abuse. Finally, the Gendercrime study discusses various theories of sexual harassment [46,47] and of women’s victimisation in particular [48–52]. Common to most of the theories outlined is the significant role that power relations play in conceptualising gender-based violence.

In terms of the target groups of the prevalence surveys, four of the studies (ARC3, HEA, KI, Wits) address both staff and students, three studies address students only (AHRC, AAU, NUS), and one study focuses on staff (UCU). In the Gendercrime study, the target group is narrowed further by addressing female students only. The FRA survey represents a special case among the ten studies regarding the target group for two reasons. First, in contrast to the other studies, it is an EU-wide survey not limited to academia. Second, the target group only includes women aged 18–74 years.

Most of the studies are also victim-focused and do not include questions about experiences of gender-based violence from a bystander and/or perpetrator perspective. In total, four of the ten studies (AAU, AHRC, HEA and KI) included prevalence questions from a bystander perspective. Only one study (KI) included questions about experiences of gender-based violence from a perpetrator perspective. All ten studies ask about the victim-perpetrator relationship and/or about the position of the perpetrator (e.g., academic staff, student), which allows the power dimension to be considered in the analysis of gender-based violence. This represents a feminist understanding of gender and violence, which defines violence as expression of power and structural inequalities.

### Determinants, individual and contextual factors

Less than half of the studies include references to theoretical conceptualisations of the measured determinants, individual and contextual factors of gender-based violence in the available technical reports (AHRC, UCU, NUS, Gendercrime). Most of them refer to measurements of previous studies and do not elaborate further on the selection criteria for including or excluding determinants, individual and contextual factors – at least not according to the information available (Table 4).

**Table 4:** Existing measurements of intersectional determinants, individual and contextual factors of gender-based violence.

ID	Study	Intersectional determinants	Individual factors	Contextual factors	Focus on individual factors	Sources used to select determinants and individual and contextual factors
1	AAU, 2015	Gender identity, student level, time at institution, age, disability, sexual orientation, ethnicity, marriage status, student living situation	Bystander behaviour, respondent’s voluntarily consumption of alcohol or drugs prior to incident, respondent’s use of alcohol or drugs without their knowledge or consent prior to incident; perpetrator’s use of alcohol or drugs prior to incident; TGQN (transgender, genderqueer, nonconforming) and first-year students named as higher risk groups	Organisational characteristics (enrolment size, public/private school, percentage of undergraduates at university, percentage of female student body); campus climate around sexual assault and sexual misconduct (including perception regarding risk of incident; knowledge and perceptions about resources, prevention trainings for new students, perceptions of responses to reporting; and bystander intervention upon suspecting or witnessing sexual assault or sexual misconduct); context prior to incident	No	Previous studies; no reference to theoretical concepts for selection of determinants, individual factors or contextual factors apparent
2	AHRC, 2017	Student level, time at institution, age, gender identity, Aboriginal and Torres Strait Islander status, cultural and linguistic background, disability, sexual orientation, field of work/study, domestic/international student, residence	Bystander behaviour, reported violence-supportive attitudes, alcohol/drug use in context of incidents of sexual violence	Knowledge about institutional policies (separate for sexual harassment and sexual assault); knowledge about provision of institutional support services (separate for sexual harassment and sexual assault)	Yes	Previous studies; no reference to theoretical concepts for selection of determinants, individual factors or contextual factors apparent
3	ARC3, 2015	Gender identity, age, sexual orientation, race, ethnicity, job category, time at institution, seniority grad, college/school	Bystander behaviour, alcohol use, hostile peer attitudes/peer norms	Institutional response	Yes	Previous studies; no reference to theoretical concepts for selection of determinants, individual factors or contextual factors apparent
4	FRA, 2014	Age, disability, immigrant/ethnic minority status, sexual orientation, education level, household composition, income, urban/rural area of living, employment status, occupation	Partner’s characteristics in case of intimate partner violence (including drinking of partner), perception of how common violence against women is; reasons for not reporting to police or other organisation/service	Contacted services of support after most serious incident, awareness of political initiatives, awareness of campaigns; EIGE Gender Equality Index; cultural differences in likelihood of reporting, in type of behaviour considered adverse social behaviour, and variations of prevalence of adverse social behaviour (verbal abuse, unwanted sexual attention, threats and humiliating behaviour, physical violence, bullying and harassment, sexual harassment)	No	Previous studies; no reference to theoretical concepts for selection of determinants, individual factors or contextual factors apparent
5	Gendercrime, 2011	Sex, age, religion, domestic/international student, time at institution, study level, field of study (faculty), residence	Use of alcohol or recreational drugs in gender-based sexual violence dating episodes, reasons for not reporting	Knowledge about provision of institutional support services	Yes	Previous studies and theoretical concepts
6	HEA, 2021	Gender, study level, work/study field, time at institution, age, sexual orientation, relationship status, ethnicity, disability, international student, residence, employment contract	Bystander attitudes and behaviour, perpetrator’s and victim’s use of alcohol and/or drugs prior to incident (students only), individual attitudes to and perceptions of sexual violence and harassment	Type of higher education institution, awareness of institutional protection/provision of support services, perception of organisational tolerance and support (handling of reporting), awareness of institutional policies and prevention measures, participation in preventative measures, feeling of safety	No	Previous studies; no reference to theoretical concepts for selection of determinants, individual factors or contextual factors apparent
7	KI, 2022	Sex, age, country of birth, foreign background, employment (classification, category, contract), field of science	Bystander behaviour	Organisational (work/study) environment	Yes	Previous studies; no reference to theoretical concepts for selection of determinants, individual factors or contextual factors apparent
8	NUS, 2018	Gender, age, race, class proxy (being the first in the family to go to university), disability, religion, sexual orientation, study level, domestic/international student	Individual behaviour on campus (professional boundaries), individual identification with institution	Institutional response	Yes	Previous studies and theoretical concepts
9	UCU, 2021	Gender identity, job category, type of contract, disability, sexual orientation	Bystander behaviour (confidence to intervene)	Confidence in institution (institutional response to reported incidents); awareness of prevention measures (training), provision of institutional support services	No	Previous studies and theoretical concepts
10	Wits, 2018	Student level, position (staff/student), age, faculty, university residence, job position/seniority grade	Sexist attitudes, attitudes towards LGBTIAQ+, alcohol/drug use in context of incidents of sexual violence	Perception of institutional protection	Yes	Previous studies; no reference to theoretical concepts for selection of determinants, individual factors or contextual factors apparent

The determining factors of gender-based violence experiences in the measurements reviewed include socio-demographic characteristics, such as sex, gender (identity), age, disability, sexual orientation, and ethnicity, which allow for intersectional analysis. However, only one of the studies (UCU) uses a theoretical concept of intersectionality. Based on the available information, it remains unclear whether and to what extent the other nine studies adopt an intersectional perspective in their measurement framework construction and if so, to what extent. In addition to socio-demographic diversity, some studies also examine aspects of functional diversity, i.e., differences in professional roles or educational levels. Common markers of functional diversity covered in the reviewed measurements are the job position, type of work contract for staff members, and study level for students.

In addition to socio-demographic characteristics and functional diversity markers, all ten studies collect data on individual factors, i.e., on individual attitudes and/or behaviours (see Table 4), which are thought to influence the prevalence of gender-based violence in universities and research organisations – or in society at large in the case of the FRA survey. Six out of ten studies examine behaviour and/or attitudes of bystanders (AAU, AHRC, ARC3, HEA, KI, UCU) and four measure attitudes, including gender beliefs, that support violence, e.g., sexist attitudes and attitudes towards LGBTIAQ+ people (AHRC, ARC3, HEA, Wits).

One thing that stood out concerning the individual factors was the strong focus on victims’ and perpetrators’ alcohol and/or drug consumption prior to incidents of gender-based violence, present in over half of the studies (AAU, ARC3, AHRC, FRA, Gendercrime, HEA, Wits). Often the analysis of this variable included arguments that it lowered interaction control on both sides: the perpetrator’s lowered self-control and the victim’s lowered self-defence control. Although, the consumption of alcohol and/or drugs may contribute to a reduction of interaction control, gender-based violence is fundamentally a structural issue of power imbalance, not merely a series of isolated incidents. For this reason, data on the role of alcohol and drug consumption in incidents must be interpreted with caution to avoid the individualisation of the structural problem of gender-based violence. Universities and research organisations need to accept their institutional responsibility of ensuring a safe work and study environment in academia [50,53,54].

Several contextual factors are considered in these studies, such as awareness or knowledge about institutional preventive measures, perception of the institutional response to reported incidents, but the review still shows a significantly stronger emphasis on individual factors in six out of the ten studies (AHRC, ARC3, Gendercrime, NUS, KI, Wits). We identify this lack of contextual factors when the studies only included general questions on the work/study environment and institutional responses, and/or addressed fewer than three Ps of the 7P-model (Prevalence, Prevention, Protection, Prosecution, Provision of Services, Policies, Partnerships) [6]. The 7P-model builds upon the conventional UN and EU 3P approach (prevention, protection, prosecution) [55] and the Council of Europe [36] Istanbul Convention’s 4P approach (prevention, protection, prosecution, policies). It provides a holistic framework for the collection of data and analysis of relationships between contextual factors and gender-based violence in universities and research organisations. In addition to questions regarding respondents’ experiences of gender-based violence (prevalence), the coverage of all 7Ps in prevalence studies entails, for example, questions on respondents’ awareness of institutional prevention mechanisms (prevention), established protection measures (protection), available institutional support services for survivors (provision of services), the existence of internal disciplinary grievance procedures or legal proceedings (prosecution procedures), the declaration of institutional intentions or strategical visions (policies), and the development of university procedures in collaboration with students, staff or their representatives. (partnerships).

It is important to consider the organisational context in which incidents of gender-based violence take place in order to gain a holistic understanding of gender-based violence, including its facilitating factors [2]. However, little attention has been paid to contextual factors so far [34]. Instead, most studies to date have focused on socio-demographic determinants or individual behavioural factors, such as alcohol consumption of victims, which are considered to heighten the risk of exposure to various forms of gender-based violence.

### Consequences of gender-based violence

Out of the ten studies, six (AAU, ARC3, FRA, Gendercrime, KI, NUS) measure consequences of gender-based violence experiences on physical and/or mental well-being. Of these six, only one (KI) does not also include questions about the impact on the respondents’ work or study satisfaction, productivity, and engagement. Another measurement of consequences that is referred to in the reviewed measurements is the respondents’ feeling of safety (ARC3, Gendercrime, HEA). Two studies (AHRC, UCU) do not include any questions concerning the consequences of gender-based violence (Table 5).

**Table 5:** Existing measurements of consequences of gender-based violence.

ID	Study	Consequences	Explicit theoretical basis	Causality burden
1	AAU, 2015	Physical and mental well-being, study-related consequences	No	Yes
2	AHRC, 2017^a^	.	.	.
3	ARC3, 2015	Physical and mental well-being, work-/study-related consequences, feeling of safety	No	Yes/No (Questions on consequences in general and as a result of past experiences of gender-based violence)
4	FRA, 2014	Physical and mental well-being, work-/study-related consequences, avoidance of specific situations due to fear	No	Yes/No (Questions on physical well-being in general, but questions on mental well-being and work-/study-related consequences “as a result” of most serious incident of past experiences of gender-based violence)
5	Gendercrime, 2011	Mental well-being, study-related consequences, feeling of safety	No	Yes/No (Questions on feeling of safety in general, but questions on physical and mental well-being, and study-related impacts as a result of past experiences of gender-based violence)
6	HEA, 2021	Feeling of safety	No	No
7	KI, 2022	Physical and mental well-being	No	No
8	NUS, 2018	Mental well-being, work-/study-related consequences, relationships with other people and with their institution	No	Yes
9	UCU, 2021^a^	.	.	.
10	Wits, 2018	Study-/work-related consequences	No	Yes

^a^AHRC (2017) and UCU (2021) did include questions on consequences in their questionnaires.

None of the ten studies refer to theoretical concepts of consequences of gender-based violence. However, the FRA survey used the results of a cognitive pre-test to distinguish between emotional responses (e.g., fear, shock, anger) and long-term psychological consequences (e.g., depression, anxiety, panic attacks) [56]. In most of the studies (six out of ten), questions on the consequences of gender-based violence are formulated as a causal link between the experience of gender-based violence and the respondents’ reported consequences, such as their impact on mental health. However, some studies vary their wording depending on the form of consequence. For instance, the FRA survey includes a general question about the respondents’ physical well-being but specifically asks about the psychological consequences resulting from the most serious incident of gender-based violence disclosed. This causal framing of consequences “as result of” is intended to establish a direct contributory link between the incident(s) of gender-based violence and their impact(s) on the respondents. Nevertheless, it is important to acknowledge that the retrospective nature of this measurement can also present methodological challenges when interpreting the data.

Two of the studies (FRA, Gendercrime) take a more comprehensive approach by asking about the consequences of the indicated incidents by form of gender-based violence. This is in contrast to most of the previous studies, which include only one or two general questions on the consequences of experiences of gender-based violence.

## Discussion

Our paper contributes to the knowledge base on gender-based violence in academia by providing a detailed overview of how existing measurements of (1) sex and gender, (2) prevalence of gender-based violence, (3) its determinants, individual and contextual factors, and (4) its consequences have been operationalised in previous prevalence studies on gender-based violence.

Taking a non-binary approach when asking respondents about their gender is a widely used practice in the measurements reviewed. However, the approaches vary considerably and some of the constructs and operationalisations of gender that are used tend to conflate the concepts of sex and gender. Experiences of violence among cis gender women are overly represented in research, reflecting normative expectations about victimisation, and neglecting the intersecting inequalities that shape experiences of gender-based violence. Empirical research suggests that it is not cis women who are most at risk of gender-based violence in the context of academia – though they are affected in large numbers, particularly by some forms of violence – but members of minoritised gender identities and/or minoritised sexual orientations. The conflation of sex and gender, in turn, is related to another conflation: that of violence against women and gender-based violence. This latter conflation is problematic in that it implicitly assumes that victims are cis women and perpetrators are cis men, without engaging with a reflection of how structural power imbalances shape the victim(s)-perpetrator(s) dynamic.

The analysis revealed another interesting finding concerning the omission of asking about the perpetrator’s(s’) gender(s) and the ordering of the categories of the gender of victim and perpetrator which speaks to the conflations discussed above. Where studies do ask about the gender of both victim and perpetrator, the order of the categories seems to reflect an implicit assumption that the victim is a woman, and the perpetrator a man. In five of the eight studies that ask for the gender of both victim and perpetrator (AAU, ARC3, Gendercrime, HEA, Wits), the first response option for victims is that of ‘woman’ or ‘female’, whereas the first response option for perpetrators is ‘man’ or ‘male’. Previous research on response biases in surveys have shown that the ordering of items matters and that different response orders may influence respondents’ answers. For example, in the context of visual presentation of response alternatives, a phenomenon known as the ‘primacy effect’ is observed. This effect describes the tendency of respondents to select one of the initial alternatives presented, rather than considering subsequent answer options. This phenomenon is particularly prevalent in self-administered surveys, such as online surveys [57]. Future studies should consider any potential bias that may be introduced by not asking for the perpetrator’s gender and/or by using a different order of the response categories for perpetrators due to primacy effect.

Our review results suggest added value of a holistic approach to measuring gender-based violence in academia, going beyond the measurement of sexual harassment and sexual violence against women, including physical, psychological, economic, and online gender-based violence against all genders. This more comprehensive approach allows for the analysis of the interrelation of different forms of gender-based violence, providing a more nuanced picture of gender-based violence in academia. Without acknowledging different forms of gender-based violence, important aspects of the phenomenon may be overlooked as the calculated prevalence and incident rates depend on the scope of the questions asked. Thus, the inclusion of some forms of gender-based violence in surveys while leaving out other ones also impacts the interpretation of prevalence rates.

While we encourage a more holistic approach beyond measuring the prevalence of sexual harassment and violence, there may be valid reasons to focus on specific forms of gender-based violence. Reducing the number of forms of gender-based violence to one or two can (1) streamline online surveys for a higher response rate, (2) contribute to designing targeted interventions, or align with legal definitions of sexual harassment and violence. The crucial aspect is to bear in mind that the chosen measurements have a direct impact on the interpretation of results. Hence, it is vital to carefully determine the survey’s objectives and identify the specific forms of gender-based violence to be assessed in advance.

Concerning the follow-up questions on experiences of gender-based violence, our position is in line with the conclusions drawn from the reviewed measurements that emphasise the importance of collecting data regarding the victim-perpetrator relationship and the role of the perpetrator, such as being an academic staff member or a student. These data are crucial for incorporating the power dynamics into an analysis of gender-based violence in its context. This is in line with a feminist understanding of gender and violence, defining violence as expression of power and structural inequalities. We argue that victims should not be portrayed as powerless but perpetrators as persons who exert violence to elevate themselves above others/to exert power over others.

Finally, when interpreting data from prevalence surveys, it is important to bear in mind the possible limitations resulting from the choice of target groups. On the one hand, this refers to the importance of addressing all people to gain a holistic understanding of gender-based violence in the respective context. On the other hand, we argue that the bystanders’ and perpetrators’ perspectives should be considered in future prevalence studies to gain a more informed estimate of prevalence.

In selecting determinants, individual and contextual factors of gender-based violence, previous studies appear to have mainly sought to establish a relationship between gender-based violence and individual-level determinants and factors. These include socio-demographic characteristics and markers of functional diversity that are thought to increase the risk of experiencing gender-based violence, as well as individual attitudes and behaviours. However, we argue that context matters and that researchers need to bear in mind what implications the prevailing focus on individual-level determinants and factors can have on the interpretation of causes and reasons of disclosed incidents of gender-based violence. An individualisation of the problem of gender-based violence can reinforce myths about gender-based violence, placing the responsibility on victims, reinforcing victimisation processes, and neglecting the structural conditions enabling gender-based violence in academic institutions. If structural problems on organisational level are individualised, systematic problems, such as hostile work environments and lack of institutional policies, remain unchallenged. In future research, contextual factors should be investigated in more detail as prevalence surveys are often used to formulate evidence-based policy recommendations. These policy recommendations for academia should be informed by not only insights on determinants and individual-level factors but also organisational ones. Institutional context can have a significant enabling or constraining role on gender-based violence at universities and research organisations.

Measurements of the consequences of gender-based violence are lacking and the question wording ‘as a result of the incident’ is often used. This approach runs the risk of using retrospective questions to establish cause-and-effect relationships between one or multiple incidents of gender-based violence and their subsequent impact on well-being and work or study. We suggest that future studies could test more extensively the extent of potential recall bias in respondents’ disclosed consequences of gender-based violence when asked retrospectively. One promising approach to this would be cognitive interviewing. The utilisation of cognitive interviews enables researchers to gain insights into how respondents interpret and recall information [58].

A limitation to the results presented is that the search for and evaluation of the measurements pursued a practical goal. Our research pursued the purpose of developing, as a next step, a questionnaire to record the prevalence of gender-based violence, including its determinants, consequences, individual and contextual factors in the context of the EU-funded UniSAFE project. In this respect, our aim in analysing previously used measurements was not to ensure overview of the full range of measurements implemented to date, but rather to identify flaws and distortions that we wanted to avoid in the design of future surveys, for example the assignment of victim and perpetrator roles according to gender identity. Our selection of measurements was also limited by including only closed-ended questions in our review and content analysis. In the meantime, first methodological tests have been carried out in which open-ended question responses from surveys in different languages are analysed with support of AI tools. As soon as these methods are fully developed, other measurement formats could be included in such a scoping review.

Our paper on the measurement of gender-based violence demonstrates how different conceptualisations and operationalisations generate evidence on gender-based violence and subsequently shape how we think and talk about the prevalence of violence in academia. This matters greatly, since what you don’t measure, you ultimately don’t see.

## Supporting information

S1 Checklist PRISMA-ScR. (PDF)
